# Supplementary material for: AffyMAPSDetector: a software tool to characterize Affymetrix GeneChip™ expression arrays with respect to SNPs
Source: BMC Bioinformatics. 2007 Jul 30;8:276. doi: 10.1186/1471-2105-8-276 (PMC1959249; doi:10.1186/1471-2105-8-276)
Supplement: Additional file 13 — AffyMAPSDetector v1 source code. AffyMAPSDetector source code. [file 1471-2105-8-276-S13.zip › AffyMAPSDetector_v1_src/doc/jwf/WizardContext.html]

WizardContext


|  |  |  |  |
| --- | --- | --- | --- |
| **Overview** \| **Package** \| **Class** \| **Use** \| **Tree** \| **Deprecated** \| **Index** \| **Help** | | |  |
| **PREV CLASS**   **NEXT CLASS** | **FRAMES**    **NO FRAMES**     **All Classes** |  |  |
| SUMMARY: NESTED \| FIELD \| CONSTR \| METHOD | DETAIL: FIELD \| CONSTR \| METHOD |  |  |


---


## jwf Class WizardContext

```
java.lang.Object
  jwf.WizardContext
```

---

public class **WizardContext** extends java.lang.Object

An instance of this class is maintained by the Wizard. WizardPanels
can store information here for use in other WizardPanels, or for the
overal purpose of the wizard.

**Author:**
:   Christopher Brind

---

**Constructor Summary**

`WizardContext()`


|  |  |
| --- | --- |
| **Method Summary** | |
| `java.lang.Object` | `getAttribute(java.lang.Object key)`             Gets an attribute. |
| `java.lang.Object` | `removeAttribute(java.lang.Object key)` |
| `void` | `setAttribute(java.lang.Object key, java.lang.Object value)`             Sets an attribute. |

**Methods inherited from class java.lang.Object**

`clone, equals, finalize, getClass, hashCode, notify, notifyAll, toString, wait, wait, wait`

**Constructor Detail**

### WizardContext

```
public WizardContext()
```


**Method Detail**

### setAttribute

```
public void setAttribute(java.lang.Object key,
                         java.lang.Object value)
```

:   Sets an attribute.

    :   **Parameters:**: `key` - an Object that is the key for this attribute: `value` - an Object this is the value of this attribute

---


### getAttribute

```
public java.lang.Object getAttribute(java.lang.Object key)
```

:   Gets an attribute.

    :   **Parameters:**: `key` - an Object used to retrieve information from this context **Returns:**: an Object

---


### removeAttribute

```
public java.lang.Object removeAttribute(java.lang.Object key)
```


---


|  |  |  |  |
| --- | --- | --- | --- |
| **Overview** \| **Package** \| **Class** \| **Use** \| **Tree** \| **Deprecated** \| **Index** \| **Help** | | |  |
| **PREV CLASS**   **NEXT CLASS** | **FRAMES**    **NO FRAMES**     **All Classes** |  |  |
| SUMMARY: NESTED \| FIELD \| CONSTR \| METHOD | DETAIL: FIELD \| CONSTR \| METHOD |  |  |


---
